# Chromosome-Scale Genome of Zoonotic Eyeworm *Thelazia callipaeda* from China

**DOI:** 10.3390/ani16172637

**Published:** 2026-08-23

**Authors:** Zichen Liu, Yang Bai, Zhenyuan Shu, Hongfei Hu, Yipeng Jin

**Affiliations:** 1 State Key Laboratory of Veterinary Public Health and Safety, Department of Clinical Veterinary Medicine, College of Veterinary Medicine, China Agricultural University, Beijing 100193, China; lzc94@126.com (Z.L.); baiyang@cau.edu.cn (Y.B.); solakc@163.com (Z.S.); 2 Guizhou Ruipailinrui Animal Hospital, Guiyang 550002, China; 18984314905@163.com

**Keywords:** *Thelazia callipaeda*, zoonotic eyeworm, chromosome-scale genome, PacBio HiFi, Hi-C, chromosome collinearity, genome annotation, pooled specimens

## Abstract

*Thelazia callipaeda* is a vector-borne zoonotic eyeworm infecting companion animals, wildlife, and humans, but chromosome-scale genomic resources from Chinese clinical material remain limited. We generated a genome supported by Pacific Biosciences (PacBio) high-fidelity (HiFi) sequencing and high-throughput chromosome conformation capture (Hi-C) from 100 adult worms recovered from naturally infected dogs in Beijing and compared its chromosome-scale organization with Portuguese assembly GCA_965194785.1. The final assembly spans 119.53 megabases (Mb) and comprises 115 top-level sequences, including four pseudomolecules totaling 91.26 Mb (76.34%) and 111 unanchored sequences. Genome-mode Benchmarking Universal Single-Copy Orthologs (BUSCO) analysis recovered 98.5% complete chromadorean orthologues, and the representative 11,788-protein gene set recovered 92.6%. Sequence-level alignment resolved Chinese chromosomes 1–4 (chr1–chr4) to Portuguese chr1, chrX, chr3, and chr2, respectively, with retained alignments covering 95.9–99.2% of each Chinese pseudomolecule and estimated sequence identities of 99.75–99.91%. Strong chromosome-scale collinearity was accompanied by localized reverse-collinear regions, including 0.243 Mb and 0.115 Mb intervals on chr2–chrX and chr3–chr3. The anchored sequences contained 96.7% of predicted genes and were substantially more gene-dense than the unanchored sequences. These results establish a clinically sourced Chinese chromosome-scale reference and provide a validated framework for future individual-worm, population-genomic, structural-variation, and comparative genomic studies of this parasite.

## Simple Summary

*Thelazia callipaeda* is a zoonotic eyeworm that infects dogs, wildlife, and people and is transmitted by tear-feeding flies. Genetic information from Chinese cases remains limited. We therefore generated a genome, representing the parasite’s complete genetic blueprint, from 100 adult worms recovered during routine treatment of naturally infected dogs in Beijing, China. Long genetic-sequence reads and information showing which genome fragments lie close together inside chromosomes were combined to assemble a 119.53-million-letter genome, of which 76.34% was organized into four large chromosome-sized sequences. Most predicted genes were located on these four sequences, and 11,788 representative genes expected to encode proteins were characterized. Direct comparison with an independently generated Portuguese genome showed clear one-to-one correspondence between the four major chromosome-sized sequences and strong overall conservation of their organization, while also identifying several small regions arranged in the opposite direction. Additional analyses showed that sequences not placed on the four major chromosome-sized sequences contained relatively few genes and quantified repeated identical sequences that may reflect the pooling of worms from multiple dogs. This validated Chinese clinical genome resource provides a foundation for future studies of parasite diversity, genome structure, transmission, and biology at individual-worm and population levels, supporting animal and public health research.

## 1. Introduction

*Thelazia callipaeda* is a spirurid nematode of veterinary and zoonotic importance whose adult stages occur principally in the conjunctival sac, beneath the third eyelid, and in associated lacrimal tissues [1,2]. The parasite infects companion animals, wildlife, and humans [1]. Transmission depends on lachryphagous drosophilid flies that ingest first-stage larvae while feeding on ocular secretions and deposit infective third-stage larvae during later feeding events [3,4]. *Phortica variegata* is the principal confirmed natural vector in Europe [5], whereas *Phortica okadai* is a confirmed vector in China and has been detected in transmission settings involving domestic and wild hosts [6]. Laboratory studies have further defined the parasite’s development in intermediate and definitive hosts [7].

The epidemiological relevance of *T. callipaeda* extends across companion-animal medicine, wildlife health, and human ophthalmology. Thelaziosis has expanded across Europe [8], and a recent survey of domestic dogs in Beijing documented spatial, seasonal, travel-related, and anthelmintic-associated differences in infection risk [9]. Human thelaziasis remains under-recognized [10], while wild carnivores may contribute to transmission networks linking wildlife, vectors, companion animals, and people [11]. These features support the value of geographically and clinically traceable genome resources for surveillance and population-level investigation.

Public *T. callipaeda* genome resources include a chromosome-scale Portuguese assembly generated from a single adult female worm [12] and an earlier Switzerland/Ticino resource distributed through WormBase ParaSite [13]. Large-scale helminth genomics illustrates the value of independently generated genome resources for comparative interpretation [14]. Together, these resources provide a framework for evaluating a clinically sourced Chinese assembly through direct same-species sequence comparison, alongside its provenance, assembly structure, annotation, and unanchored component.

Here, we characterize a chromosome-scale genome resource supported by Pacific Biosciences (PacBio) high-fidelity (HiFi) sequencing and high-throughput chromosome conformation capture (Hi-C), generated from 100 adult *T. callipaeda* pooled from naturally infected dogs treated in Beijing, China. We assess assembly continuity and gene-space completeness; compare chromosome-scale organization directly with the Portuguese assembly; describe chromosome-level gene, guanine–cytosine (GC), and repeat landscapes; summarize structural and functional annotation; and quantify exact-sequence redundancy and the unanchored component of the pooled assembly.

## 2. Materials and Methods

### 2.1. Clinical Collection, Ethics, and Pooling of Adult Worms

Adult *T. callipaeda* specimens were recovered during routine diagnosis and treatment of naturally infected dogs at the China Agricultural University Teaching Animal Hospital in Beijing, China. No dog was experimentally infected or subjected to an additional invasive procedure solely for this study. A total of 100 intact, well-preserved adult worms from multiple naturally infected dogs were selected and pooled before genomic deoxyribonucleic acid (DNA) preparation. The animal protocol was approved by the Animal Ethics Committee of China Agricultural University (approval no. AW32011202-2-1), and written informed consent was obtained from the animal owners. The assembly therefore represents pooled canine-derived clinical material from multiple naturally infected dogs.

### 2.2. Morphological Examination and Mitochondrial Species Confirmation

Recovered worms were rinsed to remove ocular debris and examined under stereomicroscopy and light microscopy. Species-level assessment considered the slender filiform body, anterior buccal and esophageal structures, sex-specific posterior morphology, and female reproductive structures. A 689 bp fragment of mitochondrial cytochrome c oxidase subunit 1 (*cox1*) was amplified using the primers NTF (5′-TGATTGGTGGTTTTGGTAA-3′) and NTR (5′-ATAAGTACGAGTATCAATATC-3′), following the established molecular characterization approach [15]. Each 50 μL reaction contained 2 μL template DNA, 2 μL of each primer (10 μmol/L), 25 μL polymerase chain reaction (PCR) premix, and 19 μL double-distilled water. The cycling conditions were 94 °C for 5 min; 35 cycles of 94 °C for 1 min, 48 °C for 1 min, and 72 °C for 1 min; and a final extension at 72 °C for 5 min. Products were examined by 1.5% agarose-gel electrophoresis, and amplicons of the expected size were subjected to bidirectional Sanger sequencing. Low-quality terminal regions were trimmed from the bidirectional sequencing reads, yielding a 564 bp high-quality consensus sequence that was used for sequence comparison. The consensus sequence was compared with published *Thelazia* sequences using the Basic Local Alignment Search Tool for nucleotide sequences (BLASTn) web service of the National Center for Biotechnology Information (NCBI) (https://blast.ncbi.nlm.nih.gov/Blast.cgi; accessed on 11 July 2026). The representative *cox1* sequence was deposited in GenBank under accession PZ737101.

### 2.3. Genomic DNA Extraction, HiFi Library Preparation, Sequencing, and Draft Assembly

High-molecular-weight genomic DNA was extracted from the pooled adult worms using the FineOut Universal Plant & Animal Genomic DNA Extraction Reagent (solution type; GENFINE Biotech (Beijing) Co., Ltd., Beijing, China). DNA integrity was assessed with the Femto Pulse system (Agilent Technologies, Santa Clara, CA, USA). A total of 6.5 μg genomic DNA was fragmented using a Megaruptor 3 (Diagenode SA, Seraing, Belgium) and purified with AMPure PB beads (Pacific Biosciences, Menlo Park, CA, USA). SMRTbell libraries were constructed using the SMRTbell Express Template Prep Kit 2.0 (Pacific Biosciences, Menlo Park, CA, USA), followed by BluePippin size selection (Sage Science, Inc., Beverly, MA, USA) to obtain an insert size of approximately 15 kb. After primer annealing and polymerase binding, sequencing was performed on the PacBio Sequel IIe platform (Pacific Biosciences, Menlo Park, CA, USA) with a 24 h run time. Provider-side processing removed low-quality and adapter-associated sequences; high-quality regions were identified with the PacBio High Quality Region Finder with signal-to-noise-ratio filtering, and circular consensus sequences (CCS) were generated from subreads using the SMRT Link ccs module (Pacific Biosciences, Menlo Park, CA, USA; https://www.pacb.com/smrt-link/; accessed on 12 July 2026). Only the resulting high-quality CCS/HiFi reads were used for assembly [16]. The sequencing report contained 10.08 Gb of HiFi bases, with a mean read length of 21.19 kb and read N50 of 21.13 kb. HiFi reads were assembled de novo with hifiasm v0.19.9 using default settings [17]. The draft assembly contained 238 contigs totaling 119.52 Mb, with a contig N50 of 1.03 Mb. Read-to-assembly mapping used minimap2 v2.28-r1209 in HiFi mode for provider-side assembly consistency assessment; the reported overall mapping rate was 99.99%.

### 2.4. Hi-C Processing and Chromosome-Scale Scaffolding

Hi-C libraries were prepared using an in situ chromosome-conformation workflow [18]. Samples were cross-linked with 1% formaldehyde for 10 min at room temperature and quenched with glycine to a final concentration of 0.125 M for 5 min. Following lysis, isolated nuclei were digested with 80 U DpnII (New England Biolabs [NEB], Ipswich, MA, USA) at 37 °C for 4 h, labelled with biotin-14-dCTP, and ligated with 4000 U T4 DNA ligase (NEB) at 20 °C. After reversal of cross-links, ligated DNA was purified with the QIAamp DNA Mini Kit (QIAGEN GmbH, Hilden, Germany), sheared to 300–500 bp, end-repaired, A-tailed, adaptor-ligated, enriched by biotin–streptavidin pull-down, PCR-amplified, and sequenced on the Illumina NovaSeq 6000 platform (Illumina, Inc., San Diego, CA, USA). Hi-C data were then used to cluster, order, and orient draft contigs into chromosome-scale scaffolds. Read mapping and duplicate-aware processing used Chromap v0.2.7-r494 [19], and artefact filtering and valid-pair classification used HiCUP v0.9.2 [20]. Valid interactions were supplied to YaHS v1.2.2 for scaffolding [21]. HiC-Pro v3.1.0 was used for contact-matrix processing and visualization [22], with the final whole-genome contact map displayed at 500 kb resolution. Hi-C processing statistics and software settings are provided in Tables S1 and S2.

### 2.5. Repeat, Structural, and Functional Annotation

Repetitive sequences were annotated with HiTE v3.3.2 [23]. Protein-coding gene prediction integrated transcriptome-supported, homology-based, and ab initio evidence, including RNA sequencing (RNA-seq) and isoform sequencing (Iso-Seq) data and protein homology from *Brugia malayi* and *Loa loa*. Evidence integration used EviAnn v2.0.3 [24], with AUGUSTUS v3.5.0 providing ab initio support [25]. The final General Feature Format version 3 (GFF3) was normalized and processed with AGAT v1.4.1 and custom scripts to retain a non-redundant representative protein-coding set of 11,788 models. Functional assignments for the representative proteins were summarized from eggNOG-mapper v2.1.13 [26] and associated Clusters of Orthologous Groups (COG), Protein families database (Pfam), Gene Ontology (GO), and Kyoto Encyclopedia of Genes and Genomes (KEGG) resources [27,28,29,30]. Annotation coverage was calculated against the 11,788-protein representative set. Gene architecture distributions were summarized for gene length, coding sequence (CDS) length, protein length, and exon number.

### 2.6. Benchmarking Universal Single-Copy Orthologs (BUSCO) Completeness and Assembly Representation

Gene-space completeness was assessed with BUSCO using the chromadorea_odb12 lineage dataset [31]. The representative-gene-set analysis was performed with BUSCO v5.8.2 and recovered 92.6% complete groups, whereas the genome-mode analysis was performed with BUSCO v6.0.0 and recovered 98.5% complete groups. The two analyses are presented separately because they use different inputs. Predicted genes, annotated repeat-covered bases, and BUSCO locations were also partitioned between anchored and unanchored sequences to quantify genomic representation by the four principal pseudomolecules.

### 2.7. Sequence-Level Comparison with the Portuguese Chromosome-Scale Assembly

The Portuguese *T. callipaeda* assembly GCA_965194785.1 (nxTheCall6.1) was obtained from NCBI. The mitochondrial molecule OZ239434.1 was excluded, leaving 104 nuclear sequences totaling 117,587,282 bp. Assembly-to-assembly alignment was performed with minimap2 v2.31-r1302 using the asm5 preset [32]. The four Chinese principal pseudomolecules were compared against Portuguese principal chromosomal molecules OZ239430.1-OZ239433.1. Panel A displays primary minimap2 chromosome alignments to visualize chromosome-scale collinearity. Quantitative chromosome correspondence and aligned-coverage values in panel B were calculated from alignments with mapping quality ≥ 20, alignment block length ≥ 10 kb, and minimap2 divergence estimate (dv) ≤ 0.02. Reciprocal CIGAR-enabled asm5 alignments were used to evaluate reverse-orientation segments, which were retained when recovered reciprocally with mapping quality ≥ 40, query span ≥ 10 kb, aligned-base identity ≥ 98%, and reciprocal coordinate overlap ≥ 50%.

### 2.8. Chromosome-Scale Genomic Landscape

GC content, predicted-gene density, and repeat coverage were summarized in non-overlapping 500 kb windows along the four principal pseudomolecules. Overlapping repeat intervals were merged before window-level unique repeat coverage was calculated. Spearman rank correlations between GC content and repeat coverage were evaluated across all windows and separately for each pseudomolecule to describe chromosome-scale covariation.

### 2.9. Exact-Sequence Redundancy and Unanchored-Sequence Characterization

CDS and protein records with exactly identical sequences within the representative annotation were grouped by sequence identity. We summarized excess identical records, their anchored or unanchored locations, duplicated BUSCO copy counts, and physical spans between anchored exactly identical CDS copies. Unanchored scaffolds were characterized by length, gene content, and unique repeat coverage. These analyses quantified repeated identical sequence records and their genomic distribution in the pooled assembly.

## 3. Results

### 3.1. Morphological and Mitochondrial Evidence Confirmed T. callipaeda

**Figure 1:**
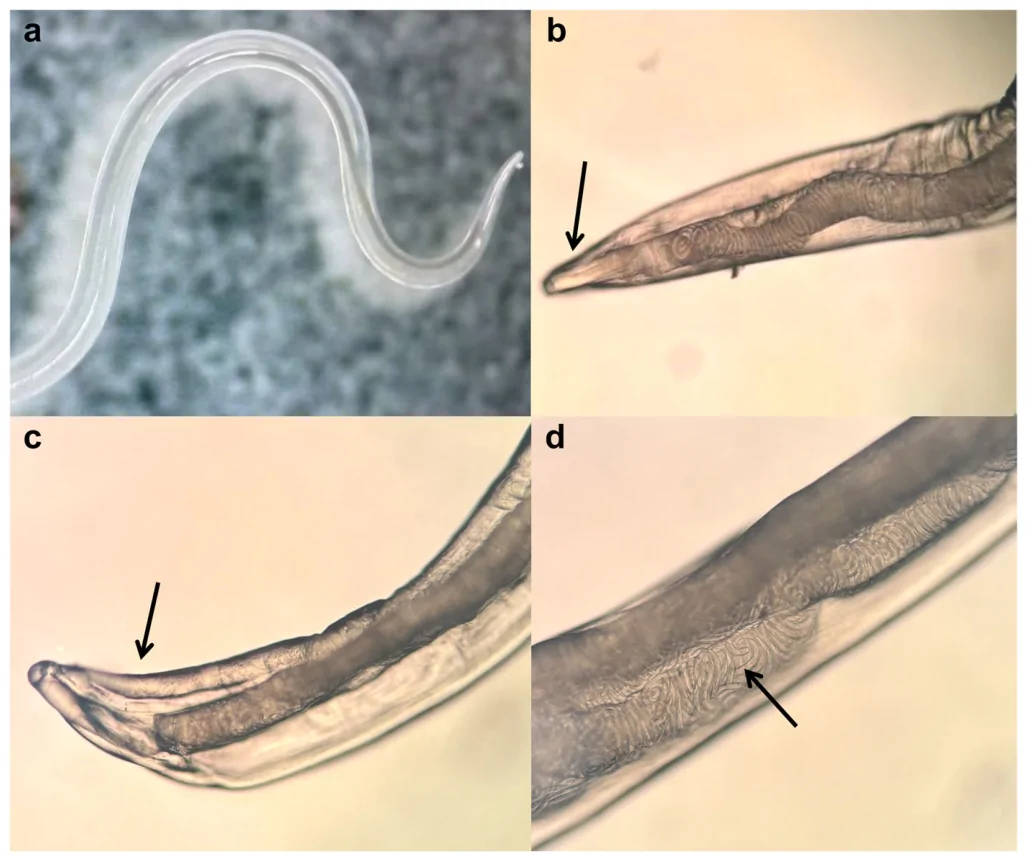
Representative morphology of adult *Thelazia callipaeda* recovered from naturally infected dogs. (**a**) Filiform adult body. (**b**) Anterior buccal and esophageal region. (**c**) Ventrally curved posterior end of an adult male. (**d**) Female reproductive tract containing intrauterine stages.

One hundred adult eyeworms recovered from naturally infected dogs in Beijing were pooled for genome sequencing. Representative specimens were slender and filiform and exhibited diagnostic morphological features, including the anterior buccal and esophageal region, the ventrally curved posterior end of an adult male, and female reproductive structures containing intrauterine stages (Figure 1). Amplification of the mitochondrial *cox1* target generated the expected 689 bp product. After trimming low-quality terminal regions from the bidirectional Sanger sequencing reads, a 564 bp high-quality consensus sequence was retained for sequence comparison and showed 99.47% nucleotide identity to a published *T. callipaeda cox1* sequence (MT040339.1). Concordance between the morphology and the mitochondrial sequence comparison supported species identification; the deposited representative *cox1* accession is PZ737101.

### 3.2. PacBio HiFi and Hi-C Data Produced a Four-Pseudomolecule Chromosome-Scale Assembly

**Figure 2:**
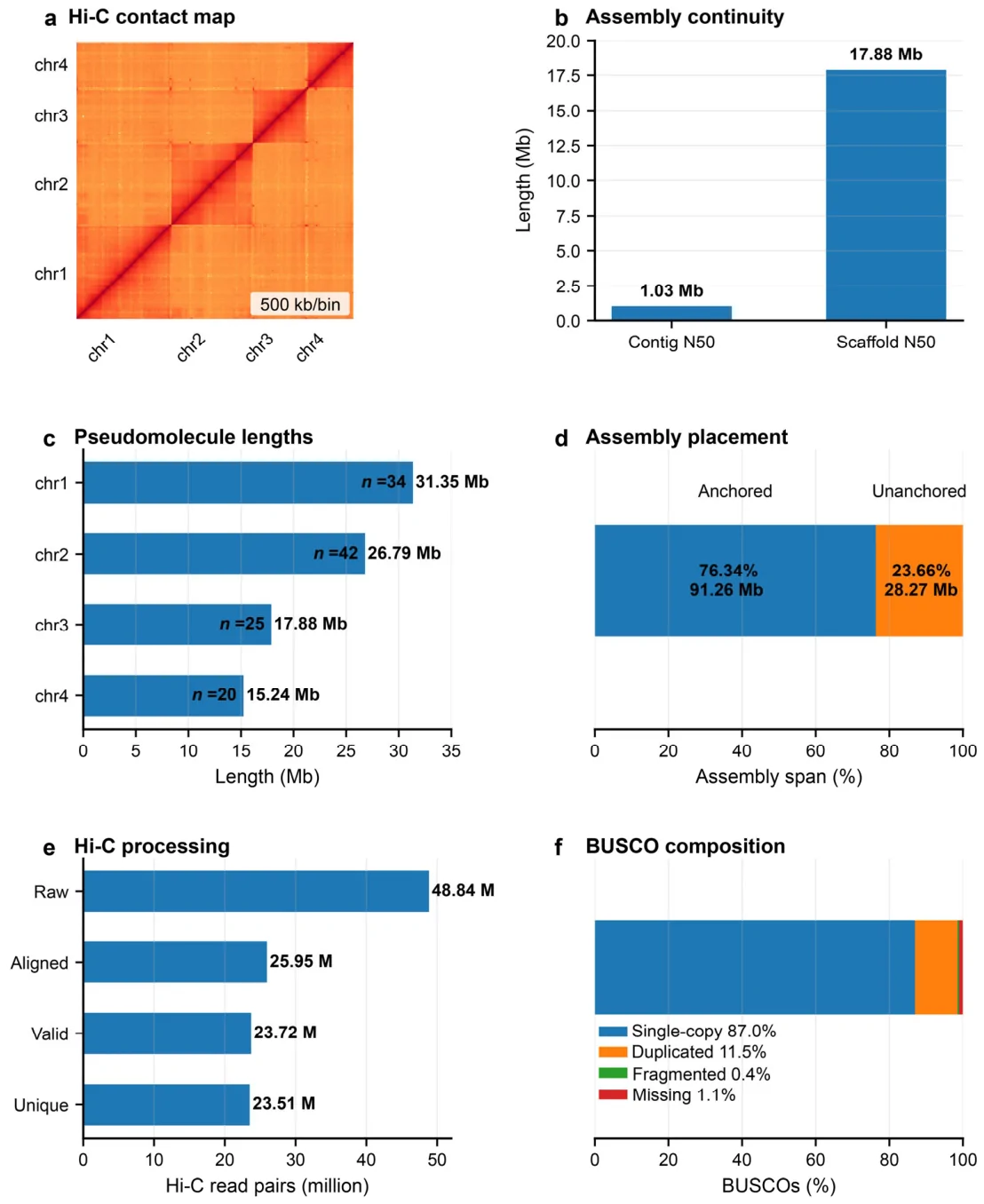
PacBio HiFi and Hi-C-supported chromosome-scale assembly of pooled Chinese *Thelazia callipaeda* resource. (**a**) Hi-C contact map at 500 kb resolution. (**b**) Contig and scaffold N50. (**c**) Pseudomolecule lengths and numbers of incorporated Hi-C input contigs. (**d**) Anchored and unanchored assembly fractions. (**e**) Hi-C processing counts. (**f**) Genome-mode BUSCO composition using chromadorea_odb12.

PacBio HiFi sequencing yielded 10.08 Gb of sequences with a long-read N50 of 21.13 kb. Following draft assembly and Hi-C scaffolding, the final chromosome-scale FASTA contained 115 top-level sequences totaling 119,534,871 bp (119.53 Mb): four principal pseudomolecules and 111 unanchored sequences. The contig N50 was 1,028,095 bp and the scaffold N50 was 17,876,072 bp. The four pseudomolecules spanned 31.35, 26.79, 17.88, and 15.24 Mb and incorporated 34, 42, 25, and 20 Hi-C input contigs, respectively. Together they represented 91,261,752 bp (76.34%) of the final assembly, whereas 28,273,119 bp (23.66%) remained unanchored (Figure 2a–d; Table 1).

Hi-C sequencing generated 48,840,474 raw read pairs; 25,953,494 formed paired alignments, 23,720,035 valid interaction pairs remained after artefact filtering, and 23,510,316 unique interaction pairs remained after deduplication (Figure 2e; Table S1). The 500 kb contact map showed four dominant intrachromosomal blocks. Genome-mode BUSCO analysis recovered 98.5% complete chromadorean groups, comprising 87.0% single-copy and 11.5% duplicated groups (Figure 2f).

### 3.3. Sequence-Level Alignment Resolved Chromosome Correspondence with the Portuguese Assembly

**Figure 3:**
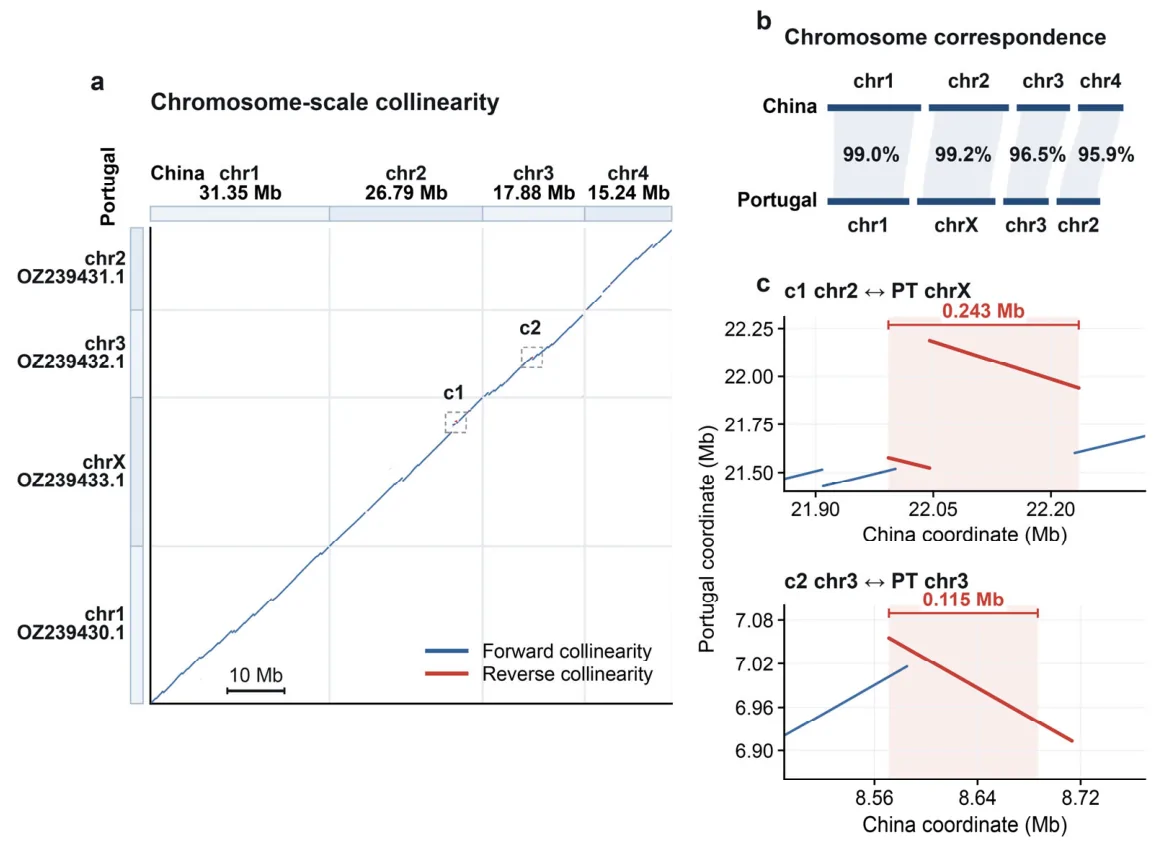
A chromosome-scale sequence comparison between the Chinese and Portuguese *Thelazia callipaeda* genome assemblies. (**a**) Sequence-level collinearity between the four principal Chinese pseudomolecules and their homologous principal chromosomal molecules in GCA_965194785.1. Forward- and reverse-orientation alignments are shown in blue and red, respectively; (**c1**,**c2**) mark regions enlarged in panel (**c**). (**b**) Sequence-supported homologous chromosome correspondence. Portuguese chromosomes are displayed in homologous order relative to the length-based Chinese pseudomolecule labels; percentages indicate the proportion of each Chinese pseudomolecule’s span covered by retained alignments. (**c**) Representative localized reverse-collinear regions: (**c1**) Chinese chr2 versus Portuguese chrX, 0.243 Mb; (**c2**) Chinese chr3 versus Portuguese chr3, 0.115 Mb.

Sequence-level alignment showed strong chromosome-scale collinearity between the Chinese and Portuguese *T. callipaeda* assemblies (Figure 3a). The length-based Chinese pseudomolecule labels corresponded unambiguously to Portuguese chr1 (OZ239430.1), chrX (OZ239433.1), chr3 (OZ239432.1), and chr2 (OZ239431.1) (Figure 3b). Retained alignments covered 99.0%, 99.2%, 96.5%, and 95.9% of Chinese chr1, chr2, chr3, and chr4, respectively (Figure 3b), with estimated aligned-sequence identities of 99.91%, 99.84%, 99.78%, and 99.75%. Forward-collinear alignments dominated all four homologous pairs, indicating broad conservation of chromosome-scale organization (Figure 3a). The broader assembly context of the Chinese, Portuguese, and Switzerland/Ticino resources is summarized in Table 2.

Reciprocal alignment also identified localized reverse-collinear segments within the otherwise strongly collinear chromosome pairs. The two largest highlighted regions spanned approximately 0.243 Mb on Chinese chr2 versus Portuguese chrX and 0.115 Mb on Chinese chr3 versus Portuguese chr3 (Figure 3(c1,c2)). Additional smaller reverse-collinear segments occurred on other homologous pairs. These intervals provide sequence-level targets for future individual-worm structural-variant analysis.

### 3.4. Anchored Pseudomolecules Captured Most Predicted Genes and Conserved Gene Space

**Figure 4:**
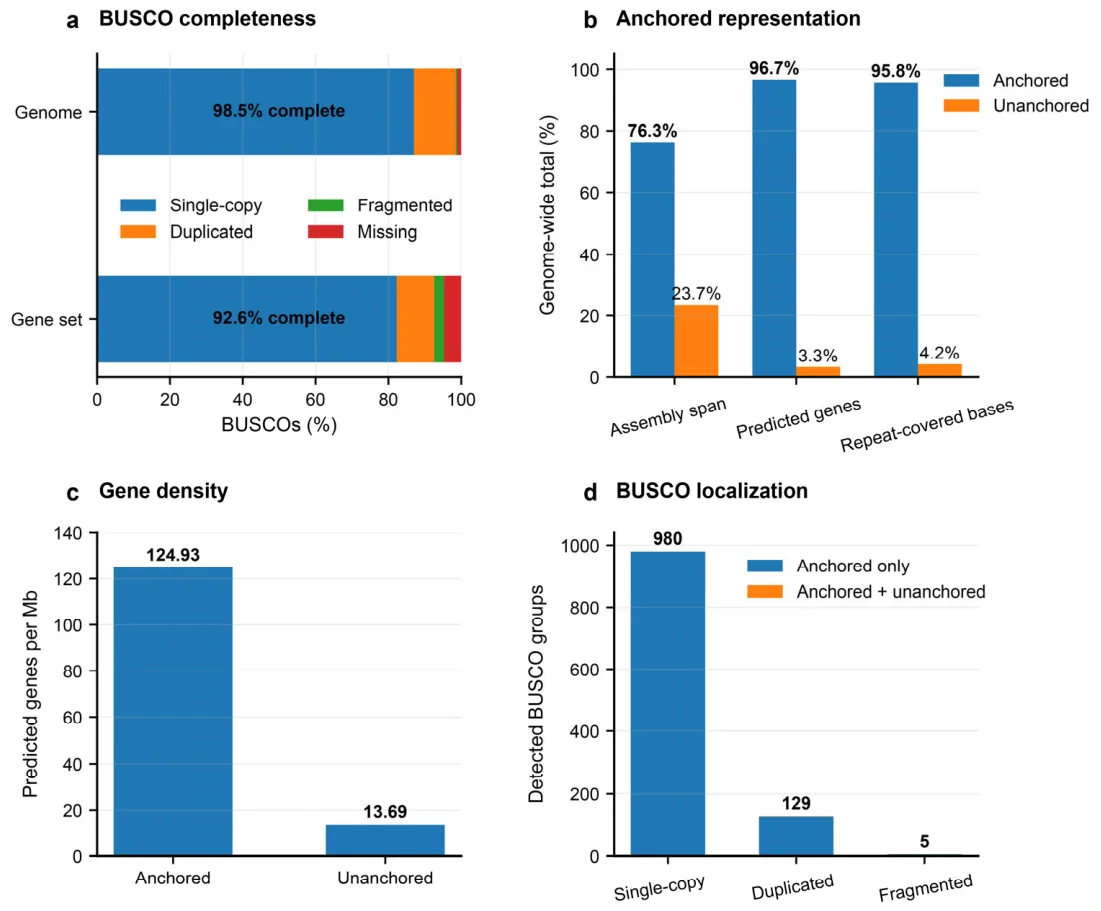
Assembly validation and genomic representation across anchored and unanchored sequences. (**a**) BUSCO completeness from the genome-mode analysis and the representative gene set. (**b**) Fractions of assembly span, predicted genes, and annotated repeat-covered bases located on anchored versus unanchored sequences. (**c**) Predicted-gene density in anchored and unanchored sequences. (**d**) Localization of detected BUSCO groups in the genome-mode analysis.

Genome-mode BUSCO analysis recovered 98.5% complete groups, whereas the representative predicted-protein analysis recovered 92.6% complete groups (Figure 4a). These estimates reflect the different analytical inputs used for the two completeness assessments. The four principal pseudomolecules represented 76.3% of the assembly span but contained 96.7% of predicted genes and 95.8% of annotated repeat-covered bases (Figure 4b). The predicted-gene density was 124.93 genes/Mb in anchored sequences compared with 13.69 genes/Mb in unanchored sequences (Figure 4c). BUSCO localization in the genome-mode analysis placed 980 complete single-copy groups and nearly all detected duplicated groups on anchored sequences; one duplicated group had copies spanning both anchored and unanchored sequences (Figure 4d).

### 3.5. Chromosome-Scale Landscapes Showed Consistent Gene-Density Patterns and a Negative GC–Repeat Association

**Figure 5:**
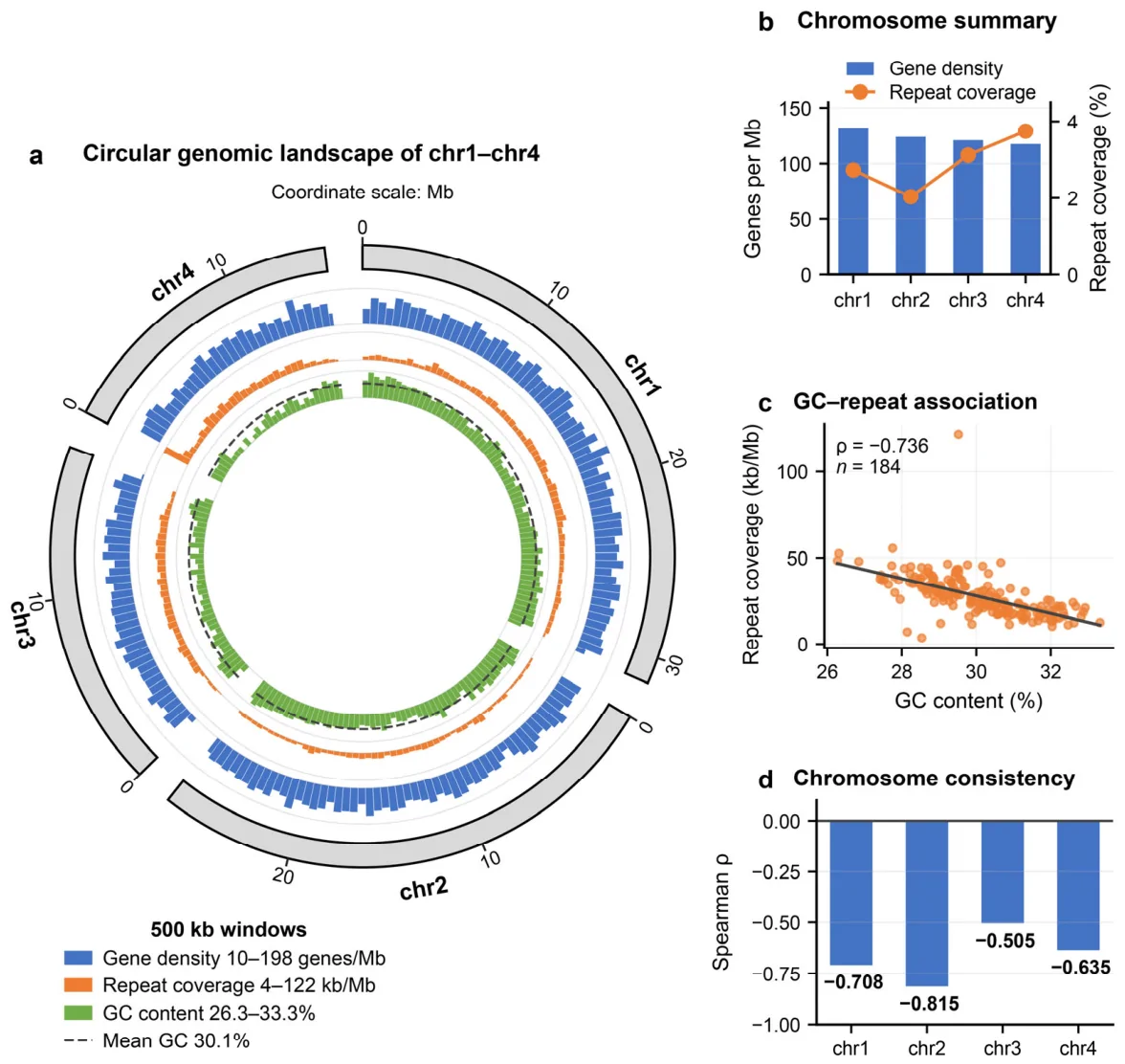
Chromosome-scale genomic landscape of Chinese *Thelazia callipaeda* assembly. (**a**) Circular genomic landscape of chr1–chr4 in non-overlapping 500 kb windows, showing predicted-gene density, unique repeat coverage, GC content, and genome-wide mean GC level. (**b**) Chromosome-level summary of predicted-gene density and unique repeat coverage. (**c**) Relationship between GC content and unique repeat coverage across 184 windows. (**d**) Chromosome-specific Spearman correlations between GC content and repeat coverage. Repeat coverage was calculated after merging overlapping repeat intervals.

Across the four principal pseudomolecules, predicted-gene densities decreased from 131.9 genes/Mb on chr1 to 117.3 genes/Mb on chr4, while unique repeat coverage ranged from 2.04% on chr2 to 3.74% on chr4 (Figure 5a,b). A window-level analysis of 184 non-overlapping 500 kb intervals revealed a strong negative Spearman association between GC content and unique repeat coverage (rho = −0.736). The association was negative on all four pseudomolecules, with chromosome-specific rho values of −0.708, −0.815, −0.505, and −0.635 for chr1–chr4, respectively (Figure 5c,d), showing a consistent chromosome-scale inverse relationship between GC content and repeat coverage.

### 3.6. The Representative Annotation Showed Broad Functional Coverage

**Figure 6:**
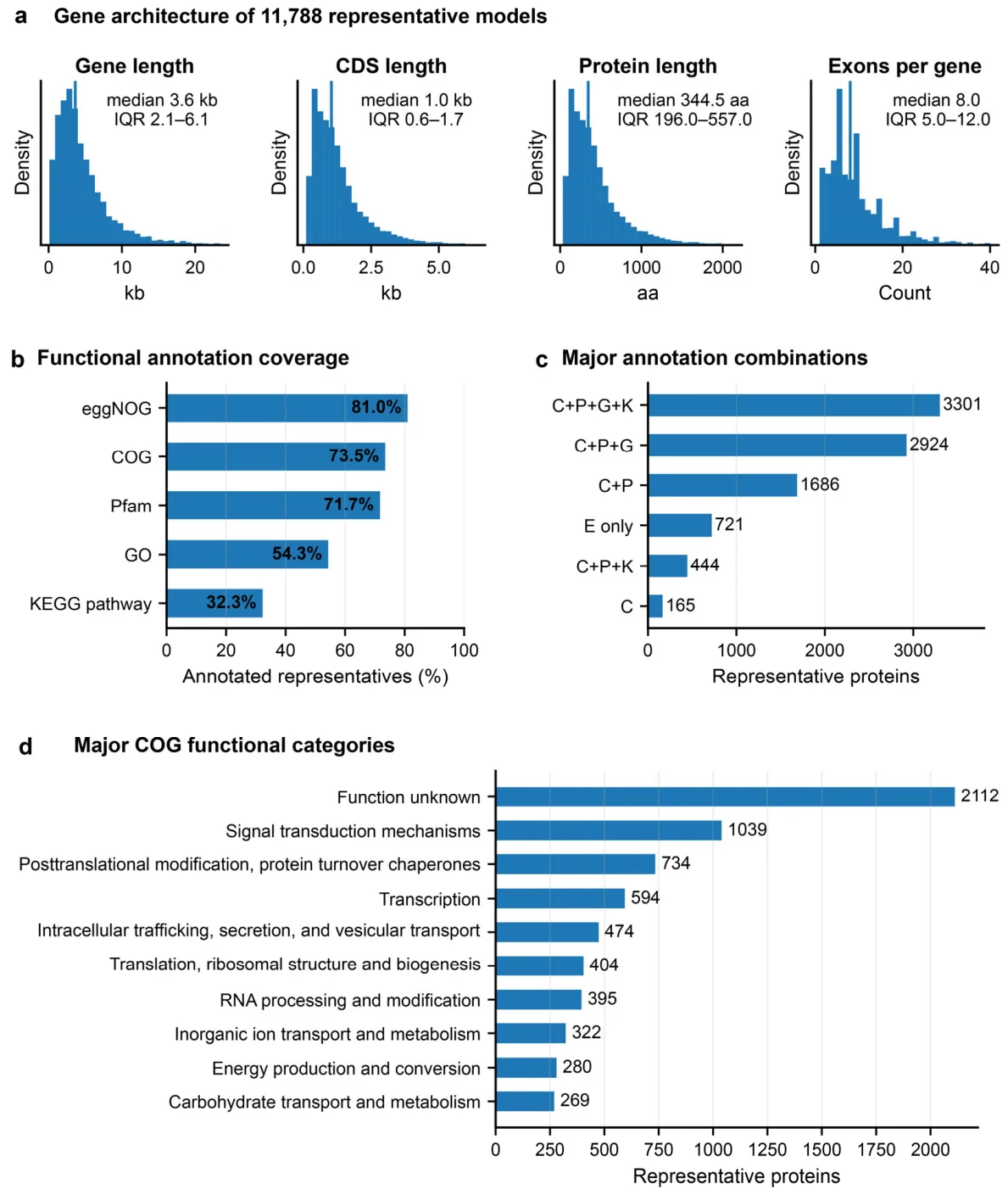
Structural and functional annotation landscape of 11,788 representative predicted models. (**a**) Distributions of gene length, CDS length, protein length, and exon count. (**b**) Functional annotation coverage across eggNOG, COG, Pfam, Gene Ontology (GO), and KEGG pathway resources. (**c**) Major annotation combinations; C, COG; P, Pfam; G, GO; K, KEGG; E only, eggNOG-only among displayed categories. (**d**) Major COG functional categories.

The representative annotation comprised 11,788 models. The median gene length was 3.6 kb (interquartile range [IQR], 2.1–6.1 kb), the median CDS length was 1.0 kb (IQR 0.6–1.7 kb), the median predicted protein length was 344.5 amino acids (aa; IQR 196.0–557.0 aa), and the median exon count was eight per gene (IQR 5–12) (Figure 6a). Among representative proteins, 81.0% received an eggNOG assignment, 73.5% a COG assignment, 71.7% a Pfam assignment, 54.3% a GO assignment, and 32.3% a KEGG pathway assignment (Figure 6b).

The largest multi-database annotation combination comprised 3301 proteins with COG, Pfam, GO, and KEGG assignments, followed by 2924 proteins with COG, Pfam, and GO assignments and 1686 with COG and Pfam assignments (Figure 6c). The most frequent COG category was function unknown (2112 proteins), followed by signal transduction mechanisms (1039) and posttranslational modification, protein turnover, and chaperones (734) (Figure 6d). Together, these distributions define the functional annotation coverage of the representative protein set and its major COG categories.

### 3.7. Exact-Sequence Redundancy and Unanchored Scaffolds

Exact-sequence analysis identified 362 excess exactly identical CDS records (3.07%) and 583 excess exactly identical protein records (4.95%) within the 11,788-record representative sets (Figure S1a). Most exactly identical groups were confined to anchored sequences: 285 of 315 CDS groups and 487 of 517 protein groups were anchored-only (Figure S1b). Among the duplicated BUSCO groups in the representative gene-set analysis, 116 had two detected copies and 13 had three copies (Figure S1c). For 285 anchored exactly identical CDS groups, the median physical span between copies was 346.5 kb (IQR 141.2–439.8 kb) (Figure S1d). These measurements quantify repeated identical sequence records within the pooled assembly and identify groups for future individual-worm and haplotype-aware analysis.

The 111 unanchored scaffolds totaled 28.27 Mb, with an N50 of 325 kb and a median length of 197 kb (Figure S2a). Thirty-eight scaffolds contained predicted genes and 73 were gene-free; gene-free scaffolds accounted for 48.6% of the unanchored span (Figure S2b). The unanchored component contained 387 predicted genes, including a small number of gene-rich scaffolds (Figure S2c). The unique merged repeat coverage was 2.21% for the whole assembly, 2.77% for anchored sequences, and 0.40% for unanchored sequences (Figure S2d).

## 4. Discussion

This study establishes a clinically traceable chromosome-scale *T. callipaeda* genome resource from naturally infected dogs in Beijing and places it in direct sequence-level context with the recently published Portuguese assembly [12]. The similar total assembly spans are complemented by clear one-to-one sequence correspondence: the four length-based Chinese pseudomolecule labels map to Portuguese chr1, chrX, chr3, and chr2. The Chinese labels were assigned by descending pseudomolecule length, whereas the Portuguese assembly uses biological chromosome labels. Forward-collinear alignments dominate all four homologous pairs, supporting broad conservation of chromosome-scale organization between the two resources.

The assembly is supported by complementary continuity, contact-map, BUSCO, and localization evidence. Hi-C scaffolding increased the scaffold N50 to 17.88 Mb and assigned 91.26 Mb to four principal pseudomolecules. Although these pseudomolecules represent 76.34% of the assembly span, they contain 96.7% of predicted genes and most of the detected conserved gene space. The 98.5% genome-mode BUSCO result and the 92.6% representative-gene-set result describe distinct analytical inputs and together provide complementary views of completeness. The remaining 28.27 Mb is comparatively gene-poor, with most of the predicted and conserved gene space represented by the anchored fraction. Future individual-worm datasets with matched high-accuracy raw reads would additionally enable reference-free k-mer assessments of base-level accuracy and completeness [33].

Whole-genome assembly comparisons provide a direct framework for localizing candidate rearrangements and local sequence differences [34]. In the Chinese–Portuguese comparison, localized reverse-collinear segments were detected within otherwise conserved homologous chromosomes, with the most prominent highlighted intervals on chr2-chrX and chr3–chr3. Their reproducibility in reciprocal alignments identifies these intervals as priorities for individual-level structural validation. Individual-worm long-read sequencing and haplotype-resolved assembly will be needed to determine whether the observed orientation differences are stable structural variants or variation among the pooled genomes.

Chromosome-scale window analysis provides a complementary view of genome organization. Gene density was relatively consistent across the four pseudomolecules, whereas repeat coverage and GC contents varied locally. The negative GC–repeat association was reproducible on all four pseudomolecules and strong in the pooled window analysis. This shared direction across chromosomes identifies a genome-wide organizational pattern that can be examined further with additional *T. callipaeda* assemblies and population-scale data.

The structural annotation and functional-coverage analysis further extend the utility of the resource. Most representative proteins received eggNOG, COG, or Pfam assignments, while GO and KEGG pathway coverage was lower. The large ‘function unknown’ COG component highlights the incomplete functional representation available for a comparatively understudied spirurid nematode. The annotation provides a foundation for gene-level follow-up, stage- and sex-resolved transcriptomics, and proteomic studies. Broad helminth genomics similarly shows that annotation depth and reference representation shape biological interpretation [14].

Pooling supplied sufficient high-quality DNA for sequencing while introducing allelic diversity from multiple worms into the assembly input. Exactly identical CDS and protein records, duplicated BUSCO copy counts, and the distribution of identical groups across anchored and unanchored sequences quantify repeated sequence records at the assembly level. Heterozygous genome assemblies can retain alternative haplotypic sequences and increase apparent redundancy [35,36,37]. Because identical records may represent paralogous, allelic, or haplotypic copies, individual-worm sequencing from defined hosts and locations, combined with read-depth and haplotype-aware analysis, will enable direct estimation of population diversity, copy-number variation, and structural polymorphism using this chromosome-scale resource as a reference.

## 5. Conclusions

This study established a clinically traceable chromosome-scale genome resource for *T. callipaeda* from naturally infected dogs in China. The genome provides a validated reference for comparative and structural genomic investigations by integrating chromosome-scale assembly, direct same-species genome comparison, and comprehensive annotation. This resource expands the genomic foundation available for this zoonotic eyeworm and will facilitate future studies on individual-worm variation, population genomics, transmission biology, and parasite evolution.

## Figures and Tables

**Table 1:** Assembly, Hi-C, annotation, and completeness metrics for the pooled Chinese *Thelazia callipaeda* genome resource.

Category	Parameter	Value
PacBio HiFi	HiFi bases	10,081,133,093 bp (10.08 Gb)
PacBio HiFi	Mean/N50 read length	21.19/21.13 kb
Genome assembly	Final assembly span	119,534,871 bp (119.53 Mb)
Genome assembly	Final top-level sequences	115 (4 pseudomolecules + 111 unanchored)
Assembly continuity	Contig N50	1,028,095 bp (1.03 Mb)
Assembly continuity	Scaffold N50	17,876,072 bp (17.88 Mb)
Hi-C	Raw/aligned read pairs	48,840,474/25,953,494
Hi-C	Valid/unique interaction pairs	23,720,035/23,510,316
Pseudomolecules	chr1	31,350,163 bp; 34 input contigs
Pseudomolecules	chr2	26,793,291 bp; 42 input contigs
Pseudomolecules	chr3	17,876,072 bp; 25 input contigs
Pseudomolecules	chr4	15,242,226 bp; 20 input contigs
Assembly placement	Anchored span	91,261,752 bp (76.34%)
Assembly placement	Unanchored span	28,273,119 bp (23.66%)
Annotation	Representative predicted proteins	11,788
Repeat annotation	Annotated repeat-feature span	2,787,503 bp (2.33%)
Repeat landscape	Unique merged repeat coverage	2,642,583 bp (2.21%)
BUSCO, genome mode	Complete groups	98.5% (87.0% single-copy; 11.5% duplicated)
BUSCO, representative gene set	Complete groups	1043/1126 (92.6%)

**Table 2:** Contextual comparison of available *Thelazia callipaeda* nuclear-genome resources.

Feature	Chinese Canine-Derived Resource	Portuguese Resource	Switzerland/Ticino Resource
Biological material	100 adults pooled from naturally infected dogs in Beijing	Single adult female worm from Portugal	Parasite material from Switzerland/Ticino
Source/accession	PRJNA1503120; JCBCPN010000000	GCA_965194785.1	GCA_900618365.1; PRJEB1205
Nuclear assembly span	119.53 Mb	117.59 Mb	75.39 Mb
Assembly context	Hi-C-supported chromosome-scale	Chromosome-scale	Scaffold-level public resource
Principal chromosome-scale molecules	4; 91.26 Mb (76.34%)	4; 83.47 Mb (70.99%)	Not reported
Chromosome-assigned unlocalized sequences	0	5.69 Mb (4.84%)	Not reported
Unplaced/unanchored sequences	28.27 Mb (23.66%)	28.43 Mb (24.18%)	Not reported
Sequence-supported correspondence	chr1->PT chr1; chr2->PT chrX; chr3->PT chr3; chr4->PT chr2	Reciprocal counterpart of Chinese mapping	Not assessed
Annotation context	11,788 representative predicted proteins	Different annotation pipeline; not directly ranked	10,912 coding genes in database annotation

The resources differ in their biological material, sequencing strategy, assembly procedure, and annotation pipeline. For GCA_965194785.1, the chromosome-assigned fraction comprises both assembled chromosome molecules and chromosome-assigned unlocalized scaffolds; these categories are reported separately.

## Data Availability

The genome assembly generated in this study has been deposited at NCBI under BioProject PRJNA1503120 and BioSample SAMN62004187. The whole-genome shotgun project accession is JCBCPN000000000, with current version JCBCPN010000000. The representative mitochondrial *cox1* sequence is available in GenBank under accession PZ737101. Additional processed datasets, intermediate analysis files, and analysis scripts that are not included in the Supplementary Materials are available from the corresponding author upon reasonable request. Public comparison assembly GCA_965194785.1 was obtained from NCBI, and the Switzerland/Ticino resource GCA_900618365.1 (BioProject PRJEB1205) was obtained through WormBase ParaSite.

## Supplementary Materials

The following supporting information can be downloaded at https://www.mdpi.com/article/10.3390/ani16172637/s1, Table S1: Hi-C mapping, filtering, and deduplication statistics for the pooled Chinese *Thelazia callipaeda* genome resource; Table S2: Software versions, datasets, and principal parameters used in genome assembly, scaffolding, annotation, sequence-level comparison, and quality-control analyses; Figure S1: Exact-sequence redundancy in the pooled assembly; Figure S2: Unanchored-scaffold architecture and repeat-coverage quality control.

## Abbreviations

The following abbreviations are used in this manuscript:

aa	amino acid(s)
BLASTn	Basic Local Alignment Search Tool for nucleotide sequences
bp	base pair(s)
BUSCO	Benchmarking Universal Single-Copy Orthologs
CCS	circular consensus sequence(s)
CDS	coding sequence
COG	Clusters of Orthologous Groups
*cox1*	cytochrome c oxidase subunit 1
DNA	deoxyribonucleic acid
GC	guanine–cytosine
GFF3	General Feature Format version 3
GO	Gene Ontology
Hi-C	high-throughput chromosome conformation capture
HiFi	high-fidelity
IQR	interquartile range
Iso-Seq	isoform sequencing
kb	kilobase(s)
KEGG	Kyoto Encyclopedia of Genes and Genomes
Mb	megabase(s)
N50	sequence length at which 50% of the total assembly length is contained in sequences of that length or longer
NCBI	National Center for Biotechnology Information
PCR	polymerase chain reaction
Pfam	Protein families database
RNA-seq	RNA sequencing
SMRT	single-molecule real-time
