# Supplementary material for: Chromosome-Scale Genome of Zoonotic Eyeworm Thelazia callipaeda from China
Source: Animals (Basel). 2026 Aug 23;16(17):2637. doi: 10.3390/ani16172637 (PMC13564313; doi:10.3390/ani16172637)
Supplement: Supplementary file 1 [file animals-16-02637-s001.zip › animals-4481296-supplementary.pdf]

Supplementary Materials

Chromosome-Scale Genome of Zoonotic Eyeworm *Thelazia callipaeda* from China

Zichen Liu[1,†], Yang Bai[1,†], Zhenyuan Shu[1], Hongfei Hu[2] and Yipeng Jin[1,*]

[1] State Key Laboratory of Veterinary Public Health and Safety, Department of Clinical Veterinary Medicine, College of Veterinary Medicine, China Agricultural University, Beijing 100193, China; lzc94@126.com (Z.L.); baiyang@cau.edu.cn (Y.B.); solakc@163.com (Z.S.)

[2] Guizhou Ruipailinrui Animal Hospital, Guiyang 550002, China; 18984314905@163.com (H.H.)

* Correspondence: yipengjin@cau.edu.cn; Tel.: +86-10-62733036

† These authors contributed equally to this work.

Supplementary Table S1. Hi-C mapping, filtering, and deduplication statistics for the pooled Chinese *Thelazia callipaeda* genome resource.

Processing stage	Category	Count	Percentage / denominator
Raw input	Read pairs	48,840,474	100%
Mapping	Paired alignments	25,953,494	53.14% of raw read pairs
Filtering	Valid interaction pairs	23,720,035	91.39% of paired alignments
Filtering	Same circularised	51,538	Excluded artefact
Filtering	Same dangling ends	299,209	Excluded artefact
Filtering	Same-fragment internal	783,500	Excluded artefact
Filtering	Re-ligation	577,812	Excluded artefact
Filtering	Contiguous sequence	278,154	Excluded artefact
Filtering	Wrong size	243,246	Excluded artefact
Deduplication	Unique interaction pairs	23,510,316	99.12% of valid interaction pairs
Interaction class	Cis-close (<10 kb)	3,006,644	12.79% of unique interaction pairs
Interaction class	Cis-far (>10 kb)	8,663,003	36.85% of unique interaction pairs
Interaction class	Trans	11,840,669	50.36% of unique interaction pairs

Percentages were calculated relative to the denominator specified for each processing stage.

Supplementary Table S2. Software versions, datasets, and principal parameters used in genome assembly, scaffolding, annotation, sequence-level comparison, and quality-control analyses.

Analysis step	Software / resource	Version	Principal setting / dataset
Genomic DNA extraction	FineOut Universal Plant & Animal Genomic DNA Extraction Reagent	Solution type	High-molecular-weight genomic DNA extraction from pooled adult worms
DNA integrity assessment	Femto Pulse	-	High-molecular-weight DNA integrity assessment before library construction
DNA fragmentation / purification	Megaruptor 3 / AMPure PB beads	-	6.5 µg genomic DNA fragmented and purified before SMRTbell preparation
HiFi library construction	SMRTbell Express Template Prep Kit 2.0 / BluePippin	-	SMRTbell library construction; size selection to approximately 15 kb inserts; primer annealing and polymerase binding
HiFi sequencing	PacBio Sequel IIe / HiFi CCS	-	24 h run; 10.08 Gb HiFi bases; mean 21.19 kb; N50 21.13 kb
Draft assembly	hifiasm	v0.19.9	Default assembly settings
HiFi read-to-assembly QC	minimap2	v2.28-r1209	HiFi read mapping; provider-side assembly QC
Hi-C library construction	DpnII / T4 DNA ligase / QIAamp DNA Mini Kit / Illumina NovaSeq 6000	-	1% formaldehyde cross-linking; 0.125 M glycine quench; DpnII digestion; biotin-14-dCTP labelling; proximity ligation; 300-500 bp library; streptavidin pull-down; PCR
Hi-C alignment / deduplication	Chromap	v0.2.7-r494	Hi-C preset; PCR duplicate removal; summary output
Hi-C artefact filtering	HiCUP	v0.9.2	Paired-end Hi-C mode; default artefact filters
Hi-C scaffolding	YaHS	v1.2.2	Default scaffolding settings
Hi-C matrix processing	HiC-Pro	v3.1.0	Valid pairs; 500 kb bins; ICE-normalized visualization
Repeat annotation	HiTE	v3.3.2	Genome-wide eukaryotic repeat annotation
Evidence-integrated gene prediction	EviAnn	v2.0.3	Evidence-integrated gene-model prediction
Ab initio support	AUGUSTUS	v3.5.0	Ab initio support incorporated into integrated annotation
GFF3 normalization / representative-model processing	AGAT + custom scripts	v1.4.1 / -	GFF3 normalization and processing to retain the non-redundant representative protein-coding set
BUSCO, representative gene set	BUSCO	v5.8.2	chromadorea_odb12; representative gene-set completeness layer
BUSCO, genome mode	BUSCO	v6.0.0	chromadorea_odb12; genome mode / miniprot sensitivity layer
Functional annotation	eggNOG-mapper	v2.1.13	Representative-protein annotations; eggNOG/COG with linked GO/KEGG assignments

Analysis step	Software / resource	Version	Principal setting / dataset
China-Portugal assembly alignment	minimap2	v2.31-r1302	asm5; MAPQ ≥20, block ≥10 kb, dv ≤0.02 for correspondence
Reverse-collinear QC	minimap2 + custom Python	v2.31-r1302 / Python 3.13.5	Reciprocal asm5 CIGAR alignments; MAPQ ≥40; span ≥10 kb; identity ≥98%; overlap ≥50%
Chromosome landscape	Custom Python workflow	Python 3.13.5	Non-overlapping 500 kb windows; merged repeat intervals; Spearman correlation
Exact-sequence redundancy	Custom Python workflow	Python 3.13.5	Exact-identical CDS/protein groups, location, copy counts, and physical span

Supplementary Figure S1. Exact-sequence redundancy in the pooled assembly.

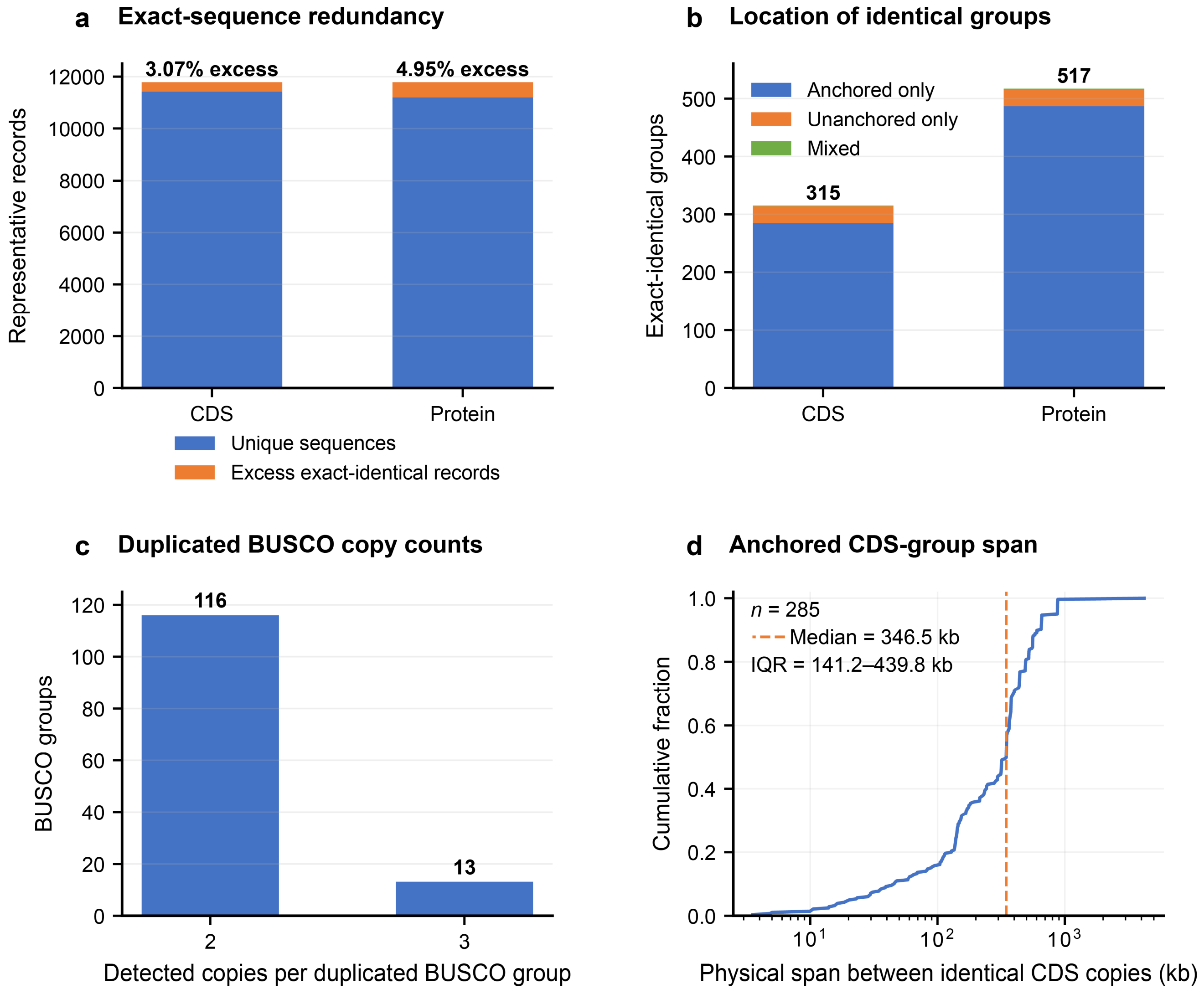

Figure S1. (a) Exact-sequence redundancy among 11,788 representative CDS and protein records. The orange component denotes excess exact-identical records beyond one representative per unique sequence. (b) Locations of exact-identical CDS and protein groups across anchored and unanchored sequence. (c) Detected copy counts among duplicated BUSCO groups in the representative gene-set analysis. (d) Cumulative distribution of physical spans between copies for anchored exact-identical CDS groups. These panels quantify repeated identical records and their genomic distribution in the pooled assembly.

Supplementary Figure S2. Unanchored-scaffold architecture and repeat-coverage quality control.

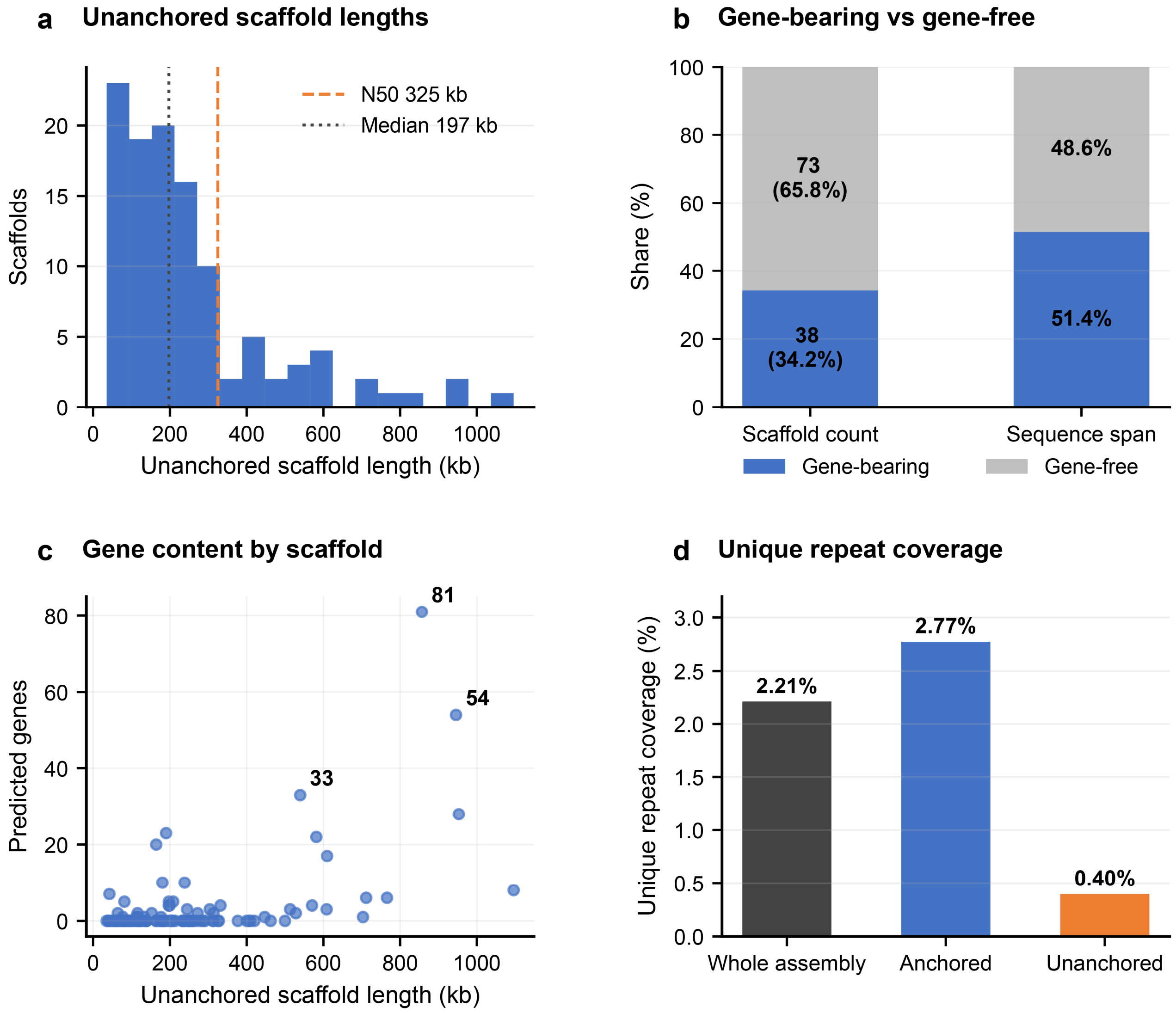

Figure S2. (a) Length distribution of the 111 unanchored scaffolds, with N50 and median length indicated. (b) Gene-bearing and gene-free scaffold fractions by scaffold count and sequence span. (c) Predicted-gene content by unanchored scaffold; labels identify the three most gene-rich scaffolds. (d) Unique merged repeat coverage for the whole assembly, anchored sequence, and unanchored sequence.
